# Feasibility of short imaging protocols for [^18^F]fluordeprenyl-D2 PET in autoimmune encephalitis and multiple system atrophy

**DOI:** 10.1007/s00259-026-07932-0

**Published:** 2026-05-28

**Authors:** Lisa Tagnin, Julia S. Dorneich, Marianthi Zeinaki, Letizia Vogler, Laura Sanzo, Johannes S. Gnörich, Sabrina Katzdobler, Ilias Masouris, Alexander Jäck, Alexander M. Bernhardt, Boris-Stephan Rauchmann, Sophia Stoecklein, Marcel Simmet, Emanuel Joseph, Simon Lindner, Norman Koglin, Andre Mueller, Andrew W. Stephens, Gérard N. Bischof, Lukas K. Frontzkowski, Nicolai Franzmeier, Rudolf A. Werner, Jonathan A. Gernert, Franziska Hopfner, Günter U. Höglinger, Robert Perneczky, Carolin Kurz, Tania Kümpfel, Martin Kerschensteiner, Franziska S. Thaler, Johannes Levin, Matthias Brendel

**Affiliations:** 1 Department of Nuclear Medicine, University Hospital of Munich, LMU Munich, Marchioninstraße 15, 81377 Munich, Germany; 2 German Center for Neurodegenerative Diseases (DZNE), Munich, Germany; 3 Department of Neurology, University Hospital of Munich, LMU Munich, Munich, Germany; 4 Department of Neuroradiology, University Hospital of Munich, LMU Munich, Munich, Germany; 5 Department of Psychiatry and Psychotherapy, University Hospital of Munich, LMU Munich, Munich, Germany; 6 Department of Radiology, University Hospital of Munich, LMU Munich, Munich, Germany; 7 Life Molecular Imaging GmbH, a Lantheus Company, Berlin, Germany; 8 Institute for Stroke and Dementia Research, LMU Hospital, LMU Munich, Munich, Germany; 9 Department of Psychiatry and Neurochemistry, Institute of Neuroscience and Physiology, The Sahlgrenska Academy, University of Gothenburg, Mölndal, Gothenburg, Sweden; 10 Russell H. Morgan Department of Radiology and Radiological Sciences, Johns Hopkins School of Medicine, Baltimore, MD U.S.A.; 11 Institute of Clinical Neuroimmunology, University Hospital, LMU Munich, Munich, Germany; 12 Munich Cluster for Systems Neurology (SyNergy), Munich, Germany; 13 Ageing Epidemiology (AGE) Research Unit, School of Public Health, Imperial College, London, UK; 14 Sheffield Institute for Translational Neuroscience (SITraN), University of Sheffield, Sheffield, UK; 15 Department of Psychiatry and Psychotherapy, Technical University of Munich, TUM School of Medicine and Health, TUM University Hospital, Munich, Germany; 16 Biomedical Center, Faculty of Medicine, LMU Munich, Munich, Germany

**Keywords:** MAO-B PET, [^18^F]fluordeprenyl-D2, Short imaging window, Autoimmune encephalitis, Multiple system atrophy

## Abstract

**Introduction:**

[^18^F]fluorodeprenyl-D2 ([^18^F]F-DED) positron emission tomography (PET) imaging detects reactive astrogliosis in patients with autoimmune encephalitis (AIE) and multiple system atrophy (MSA). Although dynamic 60-min acquisitions are established, shorter static imaging protocols are desirable for severely impaired patients. This study investigated the feasibility of short static time windows for [^18^F]F-DED PET imaging in AIE and MSA.

**Methods:**

Dynamic 60-min [^18^F]F-DED PET scans were analyzed in 20 patients with AIE, 20 patients with MSA (MSA-P/MSA-C), and 16 controls (CTRL). Disease-related lesions were manually segmented based on visually detectable positive PET-signal in AIE and MSA predilection sites (i.e. mesial temporal lobe, posterior putamen, cerebellar deep white matter), and standardized uptake value ratios (SUVr; cerebellar cortex as a reference tissue) were calculated for consecutive 10-min intervals. Advanced kinetic parameters (DVR, VTr) were derived using Logan plot and a one-tissue compartment model (1TC2k) with image-derived input functions, both applying the cerebellar cortex as a reference tissue.

**Results:**

Static images acquired between 10 and 60 min p.i. showed good image contrast and signal-to-noise ratio. SUVr of lesions increased over time and approached a plateau at approximately 50–60 min p.i.. The strongest agreement between SUVr and DVR was observed between 30 and 50 min p.i.. Late-phase SUVr outperformed kinetic parameters in discriminating lesions from healthy tissue in both AIE and MSA.

**Conclusion:**

Short static [^18^F⁸ ]F-DED PET acquisitions are clinically robust for detecting neuroinflammation in AIE and MSA. A late static acquisition between 30–50 min p.i. provides the optimal balance between accuracy and scanning efficiency.

**Supplementary Information:**

The online version contains supplementary material available at 10.1007/s00259-026-07932-0.

## Introduction

Reactive astrogliosis, characterized by morphological, molecular, and functional remodeling of astrocytes in response to injury, disease or infection of the central nervous system (CNS) [1], is a key early marker of neuroinflammation and is a joint feature of both neuroinflammatory (e.g. Autoimmune Encephalitis, AIE) and neurodegenerative diseases (e.g. Multiple System Atrophy, MSA-P/C) [2–14]. Monoamine oxidase B (MAO-B), an enzyme overexpressed on the outer mitochondrial membrane of reactive astrocytes [15, 16], is a promising biomarker target for the detection of reactive astrogliosis and is associated with neuroinflammation [17, 18]. Building on its promising results as an imaging biomarker for assessing MAO-B activity and reactive astrogliosis both in vitro and in vivo [19], this methodological study focuses on the semiquantitative and quantitative analysis of [^18^F]F-DED PET in patients with AIE and MSA-P/C. Both disease phenotypes were selected as “positive control” because their lesions are characterized by pronounced neuroinflammatory astroglial activation. In AIE, neuroinflammation is a central pathophysiological mechanism, with prominent activation of astrocytes accompanying immune-mediated neuronal dysfunction [20]. MSA is primarily classified as a neurodegenerative α-synucleinopathy, but harbors substantial neuroinflammation, including reactive astrogliosis, which has been consistently demonstrated and is thought to contribute to disease progression [21, 22]. Thus, lesions of both entities have a high likelihood of severe astrogliosis, making AIE and MSA ideal model diseases for evaluation of MAO-B-targeted PET quantification. Understanding the uptake, kinetics, and binding patterns of the MAO-B radiotracer [^18^F]F-DED PET is of clinical relevance, as it may facilitate early diagnosis, guide timely therapeutic or immunomodulatory interventions and serve as a valuable tool to monitor disease progression/course and evaluate treatment response [23].

Preliminary performance of [^18^F]F-DED using a full dynamic setting of 60-min scan has already been evaluated for the detection of neuroinflammatory astrogliosis in various diseases [24], though such long-lasting protocols remain challenging for patients and time-cost-intensive. Therefore, we aimed to investigate the suitability of short static acquisition protocols for [^18^F]F-DED MAO-B-PET imaging based on the semi-quantitative standardized uptake value ratios (SUVrs; cerebellar cortex as a reference tissue) of disease related target regions (i.e. mesial temporal lobe, posterior putamen, cerebellar deep white matter) for AIE and MSA. We hypothesized that late and short static imaging windows could provide an optimal compromise between high image quality and scanning efficiency, ultimately contributing to enhance overall patient comfort and clinical benefit. Our hypothesis was based on the observation that [^18^F]F-DED showed less irreversible binding compared to previously reported MAO-B radioligands [19], and its time-activity curves show rapid brain uptake, region-dependent washout by approximately 10–20 min p.i and stable binding in target regions during the late acquisition phase [19, 24].

To enable a more comprehensive, non-invasive assessment of [^18^F]F-DED and support both current insights and future clinical applications, we additionally quantified volume of distribution ratios (VTrs) using a one-tissue-compartment model (1TC2k) with carotid image-derived input function (IDIF) and total distribution volume ratios (DVRs) via Logan plot. In summary, we investigated 3 main hypotheses: 1) Late time windows provide a favorable balance between accuracy and economic scanning (analysis based on the semi-quantitative mean SUVr; comparison with DVR); 2) VTr with image derived input function (IDIF, carotid artery) are equivalent to DVR (Logan) and 3) Quantitative parameters (SUVr, DVR, VTr) of target regions can effectively discriminate patients from controls.

## Materials and methods

### Study design and patient enrolment

All participants were either scanned in a clinical setting or participated in observational studies of [^18^F]F-DED, including neuroinflammatory or neurodegenerative diseases. All participants or their legal representatives provided written consent for the PET imaging procedure. The study protocols and PET data analyses were approved by the local ethics committee (LMU Hospital, IRB application numbers 21–0721 and 22–0997) and the German radiation protection authorities (BfS, ZD 3–22464/2023–042-G and ZD 3–22464/2024–036-G). The study was conducted in accordance with the principles set out in the Helsinki Declaration.

**Fig. 1:**
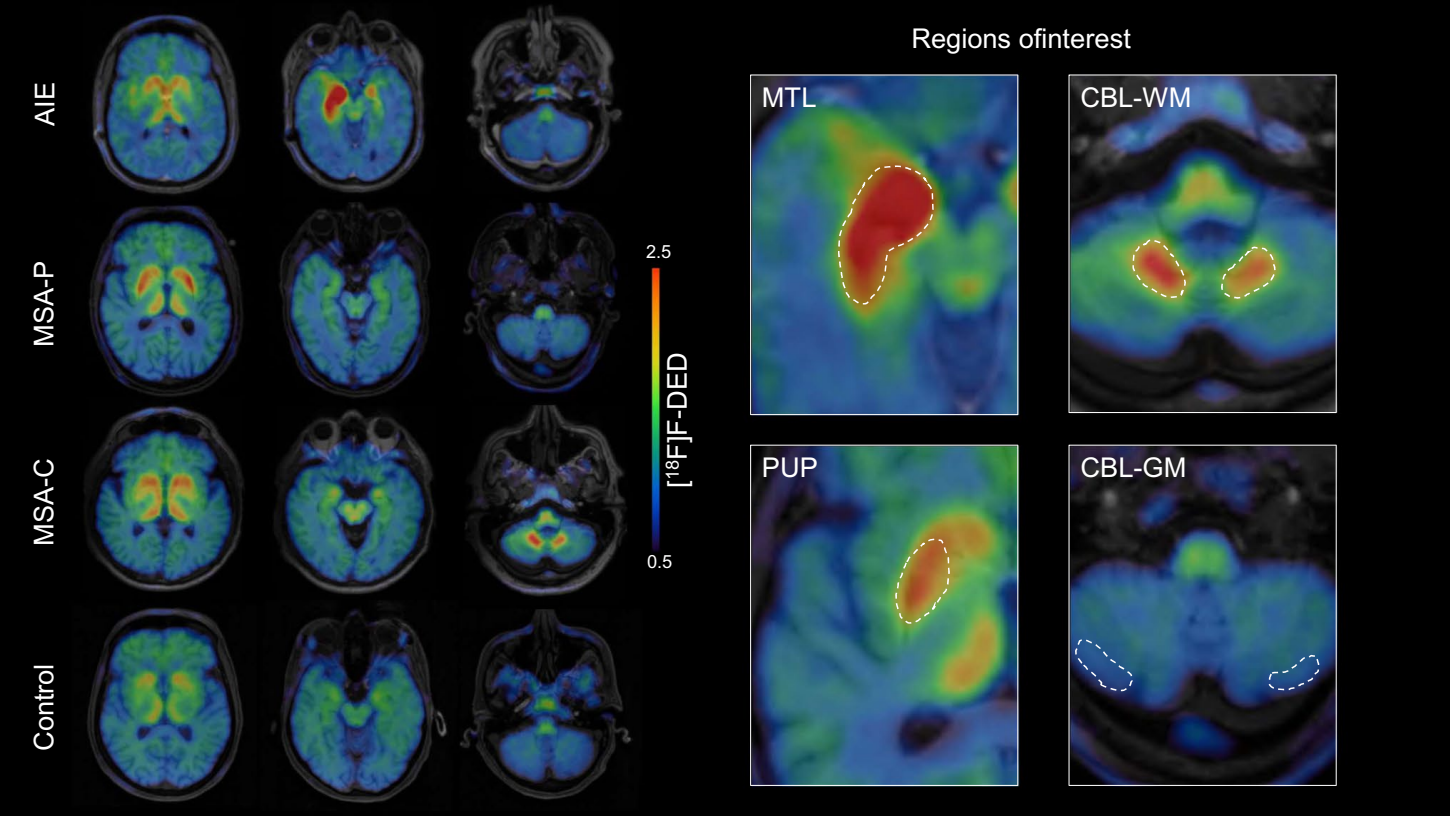
Exemplified cases and illustration of target and reference region definition. Mesial temporal lobe (MTL) for autoimmune encephalitis (AIE; anti-Ma1, anti-Ma2, anti-Ri *n* = 1, anti-LGI1, anti-DPPX, anti-CASPR2, anti-GAD65, anti-Rho, seronegative), posterior putamen (pPUT) and cerebellar white matter (CBL-WM) for multiple system atrophy, either Parkinsonian (MSA-P) or cerebellar (MSA-C) subtype. The control shows typical regional distribution of MAO-B expression, which is highest in the basal ganglia and thalamus, followed by the basal forebrain and substantia nigra. Intermediate levels are found in the anterior cingulate, gyrus rectus and hippocampus, whereas lowest levels occur in neocortical areas, the subcortical white matter and the cerebellar cortex. Regions of interest are outlined by white dashed lines. The cerebellar gray matter (CBL-GM) served as the reference region. [^18^F]F-DED PET images of the 30–60 min p.i. time window are shown as three axial layers upon the individual T1 MRI

We randomly selected 20 patients with clinically possible/established AIE (anti-Ma1-, anti-Ma2- and anti-Ri-associated AIE n = 1, anti-LGI1-associated limbic encephalitis n = 3, anti-LGI-1-associated AIE *n* = 4, anti-DPPX-associated AIE *n* = 1, anti-LGI1- and anti-CASPR2-associated AIE *n* = 1, anti-GAD65-associated limbic encephalitis *n* = 1, anti-Rho-associated AIE *n* = 1, anti-GAD65-aassociated AIE *n* = 3, suspected anti-GAD65-associated TLE *n* = 1, seronegative AIE *n* = 3, anti-GAD65-associated cerebellar ataxia *n* = 1) according to Graus-criteria for subacute courses [25] and GAD-specific criteria for GAD-positive encephalitis [26] from a cohort of 111 patients with AIE. [^18^F]F-DED PET data on anti-LGI1 AIE has already been published [23]. Furthermore, 20 patients with clinically probable/established MSA (MSA-P *n* = 12, MSA-C *n* = 8) according to MDS- [27] and Gilman-criteria [28] were selected from a cohort of 36 patients with MSA. A visually positive rating of at least one [^18^F]F-DED-positive predilection site (i.e. mesial temporal lobe, posterior putamen, cerebellar deep white matter) during clinical assessment was defined as inclusion criteria with the rationale to test different time-windows in lesions with reactive astrogliosis (Fig. 1). All available *n* = 16 controls were selected from our local database [23] and equivalent regions were subject of analysis. Inclusion criteria for controls included a mini-mental-state examination score ≥ 27 and negative testing for biomarkers of β-amyloid and tau. All controls except one individual with a small oligodendroglioma (outside the target regions) had no objectified diagnosis after clinical evaluation. Exclusion criteria were psychiatric disorders (e.g., addiction, severe psychosis, depression with BDI > 18 points) and relevant internal medical conditions (e.g., severe renal and hepatic insufficiency, heart failure, coronary heart disease, septic conditions, uncontrolled diabetes mellitus).

### PET imaging

#### Radiosynthesis

[^18^F]F-DED was synthesized in-house in the Department of Nuclear Medicine and Radiopharmacy of the LMU Hospital, Munich as described previously [24].

#### PET acquisition and preparation

All patients and participants were scanned at the Department of Nuclear Medicine, LMU Munich, with a Biograph 64 or a Siemens mCT PET/CT scanner (both Siemens, Erlangen, Germany). Caffeine consumption was prohibited 3 h prior to tracer injection. Active smokers were excluded from [^18^F]F-DED PET imaging. A low-dose CT-scan preceded the PET-acquisition and served for attenuation correction. [^18^F]F-DED PET was performed in a full dynamic 0–60-min setting initiated upon intravenous injection (~ 10 s) of 174 ± 21 MBq [^18^F]F-DED (range, 120–206 MBq [^18^F]F-DED), followed by a 10 to 20 ml saline flush. OSEM3D reconstruction was performed as previously described [29]. [^18^F]F-DED PET data were reconstructed in a series of 35 frames (i.e., 12 × 5 s, 6 × 10 s, 3 × 20 s, 7 × 60 s, 2 × 300 s, 3 × 600 s) and also binned into a single static frames of 10-min duration (*see* below) as well as a frame ranging from 30 to 60 min p.i. for initial clinical evaluation/visual assessment. All dynamic images were checked visually and, if necessary, automatically corrected for head motion using PMOD.

#### Image pre-processing

All image data were processed and analyzed using HERMIA GoldLx (version 2.17.0.4, Hermes Medical Solutions AB, Stockholm, Sweden) and PMOD (version 3.5, PMOD Technologies Ltd., Zurich, Switzerland). For spatial normalization, a tracer-specific template in the MNI space was created for the 30–60 min [^18^F]F-DED frame. Specifically, the mixed-cohort template was generated by averaging the scans of n = 8 controls, n = 8 MSA-C patients, and n = 8 AIE patients, that were spatially normalized via a T1 MPRAGE MRI with the PNEURO tool. Dynamic [^18^F]F-DED images were co-registered to the MNI space by applying the 30–60-min transformation (brain normalization settings: nonlinear warping, 8-mm input smoothing, equal modality, 16 iterations, frequency cut-off 3, regularization 1.0, no thresholding).

### PET data evaluation/PET data analysis and visual inspection

#### Definition of volumes of interest (VOIs) and visual assessment

Volumes of interest (VOIs) were defined manually based on both disease-specific topology and qualitative visual assessment. Target regions were identified according to visually detectable PET-positive signals; mesial temporal lobe (MTL, *n* = 18), temporopolar cortex (*n* = 1) and frontal cortex (*n* = 1) in AIE and the posterior putamen (pPUT, *n* = 14) or cerebellar white matter (CBL-WM, *n* = 6) in MSA-P/C, as shown in Fig. 1.

To ensure a robust (semi)quantification of reactive astrogliosis in target regions, the cerebellar gray matter (GM) was manually defined and used as the reference region (Fig. 1). The reference region was consistently placed within the posterior or superior cerebellar lobe, either in the lateral hemisphere or the intermediate zone, with only low inter-individual variation due to anatomical differences. This region was selected due to its consistently low MAO-B expression and minimal [^18^F]F-DED uptake, as reported in both preclinical and clinical studies [24, 30, 31]. Although MAO-B is ubiquitously expressed throughout the brain, predominantly in reactive astrocytes [5, 15] and monoaminergic neurons, and to a lesser extent in oligodendrocytes, microglia, and endothelial cells [32], its regional distribution is lowest in the cerebellar cortex [18, 33, 34]. Furthermore, cerebellar GM showed stable [^18^F]F-DED binding across disease states and age groups [24], making it a widely accepted pseudo-reference region in MAO-B PET imaging. Suitability of the cerebellar GM as a reference region for the analyzed dataset was determined by SUV comparison between controls, and patients with AIE and MSA, across all time windows using Welch’s t-test. Comparisons showed no significant differences for AIE vs. CTRL and MSA vs. CTRL after False Discovery Rate (FDR) correction (all p_adj_ ≥ 0.14; Supplemental Fig. 1) supporting the use of the cerebellar GM as a reference region. Accordingly, it was used for normalization in both semi-quantitative and quantitative analyses. Separation of cerebellar white matter and gray matter was performed manually using the anatomical boundaries visible on the individual MRI images or MRI atlas template as guidance.

To mimic a clinical scenario, visually positive cortical and subcortical lesions were first identified and manually delineated in HERMIA GoldX (version 2.17.0.4, Hermes Medical Solutions AB, Stockholm, Sweden) using freehand regions of interest. The cerebellar gray matter reference region was likewise identified manually. Subsequently, we used PMOD (version 3.5, PMOD Technologies Ltd., Zurich, Switzerland) as a state-of-the-art processing tool for scientific purpose. Here, PET values were extracted using a standardized spherical VOI (5 mm diameter) placed within the manually identified lesion. For the reference region, PET values were extracted from a standardized spherical VOI (7 mm diameter) placed in the cerebellar gray matter.

For the comparison of patients lesions against healthy tissue in controls, corresponding regions of interest were additionally placed in the mesial temporal lobe, in the posterior putamen and in the cerebellar grey matter of controls.

#### Extraction of semi-quantitative parameters

The full dynamic datasets (0–60 min p.i.) were binned into a series of 10-min intervals, visually showing a good image contrast and strong signal to noise ratio (Supplemental Fig. 2). SUVr values were calculated with the cerebellum as a reference tissue, extracted from the corresponding static images (0–10, 10–20, 20–30, 30–40, 40–50, and 50–60 min p.i.) using HERMIA GoldLx (version 2.17.0.4, Hermes Medical Solutions AB, Stockholm, Sweden). Parametric images (VTr and DVR) were generated from the full 0–60 min dataset using PMOD (Version 3.4, PMOD Technologies Ltd., Zurich, Switzerland) according to previously established workflows [24], employing a one-tissue compartment model (1TC2k) for VTr estimation with a carotid image-derived input function [35, 36] and the graphical reference tissue-based Logan plot for DVR calculation [37]. The plasma input curve was obtained from a standardized spherical VOI (5 mm diameter) placed manually in the carotid artery on the early PET images, where the vessel was most clearly visualized, as described previously [38]. A maximum error of 10% and a VT threshold of 0 were selected for modelling of the full dynamic imaging data. SUVr, VTr, and DVR values were submitted to further statistical analyses of [^18^F]F-DED PET data. Representative [^18^F]F-DED mean SUVr images of all different time windows are shown in Fig. 2.

**Fig. 2:**
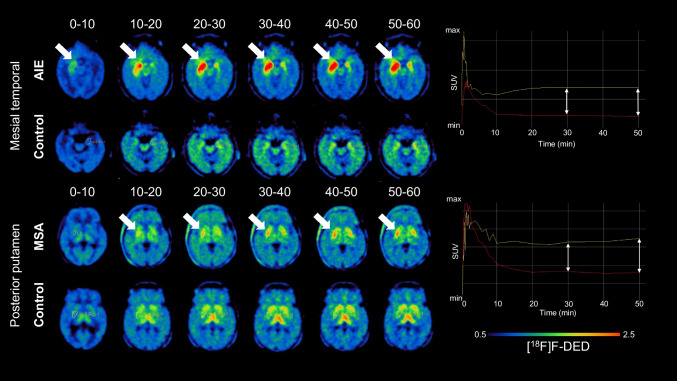
Trajectories of relative [^18^F]F-DED uptake during 60 min scan duration. Upper two rows show [^18^F]F-DED SUVr images in the axial plane of the mesial temporal lobe (MTL) for one patient with autoimmune encephalitis (AIE) in comparison to one control. Lower two rows show [^18^F]F-DED SUVr images in the axial plane of the posterior putamen (pPUT) for one patient with multiple system atrophy (MSA) in comparison to one control. Each image represents a single 10-min frame. Time-activity-curves on the right show SUV in target (yellow) and reference (red) regions as a function of time (50 min indicate the start of the last frame). PET-positive target lesions are indicated by the white arrows

## Statistical analysis

The following quantitative comparisons between patients with AIE, MSA and controls were performed: (I) As the main analysis, mean SUVr of all time windows were compared within the cohorts of AIE and MSA patients using a mixed ANOVA Test with Tukey´s correction and calculation of effect sizes (Cohen’s d). (II) SUVr of all time windows and DVR were correlated for AIE and MSA with a linear regression; (III) SUVr, DVR and VTr of mesial temporal lesions (*n* = 18) and posterior putamen lesions (*n* = 14) were compared to corresponding healthy tissue of controls using multiple t-tests with False Discovery Rate correction.

**Fig. 3:**
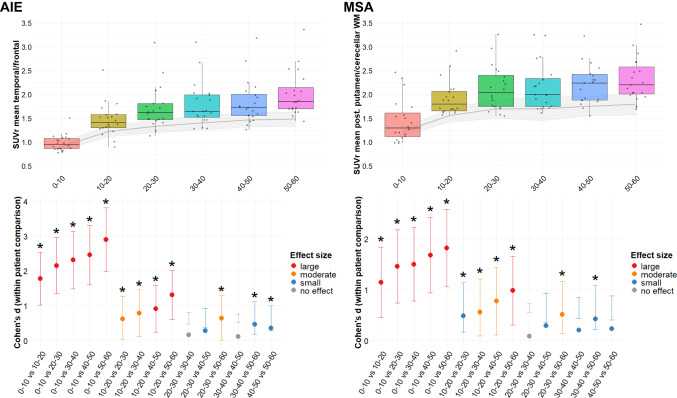
Quantitative comparison between lesion SUVr in different 10-min intervals for [^18^F]F-DED PET. Simplified image quantification (SUVr) of the [^18^F]F-DED PET signal in comparison of different time windows (0–10, 10–20, 20–30, 30–40, 40–50, 50–60 min p.i. in lesions of patients with autoimmune encephalitis (AIE, *n* = 20) and multiple system atrophy (MSA, *n* = 20) is shown by box plots together with effect size comparison between different time windows (Cohen´s d). A mixed ANOVA Test with Tukey´s post hoc correction was used for statistical comparisons. The gray reference line and surrounding shaded area represent the SUVr mean ± standard deviation of the control group

For the comparison of SUVr mean values among the 10-min intervals for AIE and MSA, assumptions of normality were checked with Shapiro–Wilk test, the Kolmogorov–Smirnov test, and visual inspection of Q–Q plots. In the analysis displayed in Fig. 3, mean SUVr of all time windows were compared within the cohorts of AIE and MSA patients using a repeated-measures analysis implemented via a linear mixed-effects model, reflecting a mixed-design ANOVA structure. 10-min intervals were included as a within-subject factor, and PET SUVr values served as the dependent variable. To account for repeated measurements within individuals, subject-specific random intercepts (Subject_ID) were included as random effects. Post-hoc pairwise comparisons between time intervals were adjusted using Tukey’s correction to control for multiple comparisons and reduce the risk of Type I error. Effect sizes for these comparisons were quantified using Cohen’s *d* with a standardized measure of the magnitude of the observed differences as described in the results section: no effect |d|< 0.20, small 0.20 ≤|d|< 0.50, moderate 0.50 ≤|d|< 0.80, and large |d|≥ 0.80).

Linear regression was used to assess the association between mean SUVr across all time windows and DVR. The assumptions of linearity and homoscedasticity were evaluated separately for each cohort. In the AIE cohort, both assumptions were met, allowing the use of standard linear regression. In the MSA cohort, some SUVr values violated the homoscedasticity assumption, and therefore robust linear regression was applied.

To compare PET measures between lesioned regions and corresponding healthy tissue in controls, multiple independent t-tests were performed for SUVr, DVR, and VTr values in mesial temporal lesions (*n* = 18) and posterior putamen lesions (*n* = 14). Normality was assessed using the Shapiro–Wilk test and Q–Q plots, and homogeneity of variance was evaluated with Levene’s test. To account for multiple comparisons, p-values were adjusted using the False Discovery Rate (FDR) method, which controls the expected proportion of false positives while maintaining statistical power and is preferable to overly conservative corrections, such as Bonferroni, when several tests are performed.

A significance level of *p* < 0.05 was applied in all analyses. All statistical analyses were performed using R Version 4.4.1 [39].

## Results

### Demographics

A total of 56 patients and participants were included in the analysis. The cohort consisted of 20 patients with AIE (age 52.3 ± 17.8, male/female 13/7), 20 patients with MSA-P/C (age 60.3 ± 7.0, male/female 14/6) and 16 controls (age 67.9 ± 15.2, male/female 7/9). All groups did not differ in sex (AIE vs MSA: *p* = 0.7; patients vs CTRL: *p* = 0.1, Χ^2^-test) and the patient cohorts did not differ in age (*p* = 0.07, t-test). Controls were significantly older than patients (all patients vs CTRL: *p* = 0.008, t-test; AIE vs CTRL: *p* = 0.009, MSA vs CTRL: *p* = 0.05). Demographics are detailed in Table 1.

**Table 1:** Demographic representation of all participants

	AIE *n* = 20	MSA *n* = 20	CTRL *n* = 16	*p*-value (patients vs CTRL)	*p*-value (AIE vs CTRL)	*p*-value (MSA vs CTRL)
Age, years (mean ± SD)	52.3 ± 17.8	60.3 ± 7.0	67.9 ± 15.2	0.008	0.009	0.05
Female sex, *n* (%)	7 (35.0%)	6 (30.0%)	9 (56.3%)	0.1	0.2	0.11
AIE antibody, *n* (%)						
Anti-Ma1-, anti-Ma2- and anti-Ri	1 (5.0%)	-	-			
Anti-LGI1	7 (35.0%)	-	-			
Anti-LGI1- and anti-CASPR2	1 (5.0%)	-	-			
Anti-DPPX	1 (5.0%)	-	-			
Anti-GAD65	6 (30.0%)	-	-			
Anti-Rho	1 (5.0%)	-	-			
Seronegative	3 (15.0%)	-	-			
MSA-subtype, *n* (%)						
Parkinsonian subtype	-	12 (66.7%)	-			
Cerebellar subtype	-	8 (33.3%)	-			
Referral of controls						
Suspected but not objectified memory complaints	-	-	13 (81.3%)			
Suspected systemic inflammation	-	-	1 (6.35%)			
Suspected Parkinson´s disease	-	-	1 (6.35%)			
Oligodendroglioma	-	-	1 (6.35%)			
Target lesions						
Mesiotemporal Lobe (MTL)	18 (90.0%)	-	-			
Frontal	1 (5.0%)	-	-			
Temporopolar	1 (5.0%)	-	-			
Posterior Putamen	-	14 (70.0%)	-			
Cerebellar white matter	-	6 (30.0%)	-			

Values are presented as number of cases (*n*), mean ± standard deviation (SD), percentage (%), as appropriate. *AIE* autoimmune encephalitis, *MSA* multiple system atrophy, *CTRL* controls. Age and sex differences were analyzed using t-test (age) and Χ^2^-test (sex)

### [^18^F]F-DED SUVr of AIE and MSA lesions across different time windows

Mean SUVr showed significant differences over the 60-min dynamic acquisition in both AIE and MSA lesions (Fig. 2). Lesion SUVr increased rapidly in the early-phase of the scan (0–20/30 min p.i.), followed by stabilization from approximately 30 min onward. This trend was also evident in the time-activity curves, where SUVr values began to stabilize around 30 min p.i. and approached an apparent steady state between 50 and 60 min. Quantitatively, mean SUVr values of later time windows (20–30, 30–40, 40–50, and 50–60 min p.i.) were significantly higher compared to early time windows (0–10 and 10–20 min p.i.). A detailed visualization of [^18^F]F-DED mean SUVr distributions and effect sizes across time windows is provided in Fig. 3. Effect sizes were quantified using Cohen’s d and interpreted according to conventional thresholds (no effect |d|< 0.20, small 0.20 ≤|d|< 0.50, moderate 0.50 ≤|d|< 0.80, and large |d|≥ 0.80) [40]. For AIE lesions, all comparisons showed highly significant differences (*p* < 0.001) with moderate to large effect sizes (Cohen’s d: 0.6–2.9). Similarly, in MSA lesions, significant differences were also observed across all comparisons (*p* < 0.05) with moderate to large effect sizes (Cohen’s d: 0.5–1.8). A further small increase in mean SUVr was detected between 40–50 and 50–60 min p.i. windows in AIE lesions (*p* = 0.024, Cohen’s d: 0.4), whereas SUVr of MSA lesions already reached a plateau. SUVr of the analyzed target regions in controls approached a plateau towards 60 min p.i..

Statistical comparisons, including mixed ANOVA, Tukey post-hoc tests, and Cohen’s d are summarized in Supplemental Tables 1, 2, 3 and 4.

### Agreement between [^18^F]F-DED SUVr and DVR across static time windows

**Fig. 4:**
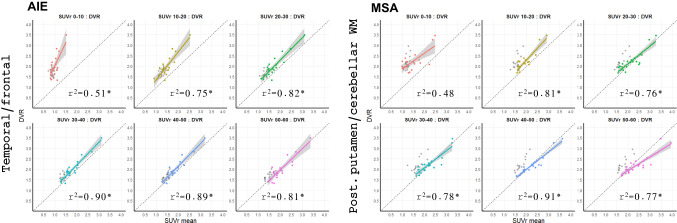
Agreement of simplified quantification with reference tissue modelling. Correlation of [^18^F]F-DED PET visual positive signals derived from lesions of patients with autoimmune encephalitis (AIE, *n* = 20; MTL = 18, frontal = 1, temporopolar = 1) and multiple system atrophy (MSA, *n* = 20; pPUT = 14, CBL-WM = 5, brainstem = 1) between standardized uptake value ratios (SUVr; 0–10, 10–20, 20–30, 30–40, 40–50, 50–60 min p.i.) and reference tissue modeling (DVR) of the full dynamic PET scan. Dashed line corresponds to perfect agreement (y = x). Linear regression served for calculation of r^2^. * = *p* < 0.001. Control values are plotted in dark gray

Next, we asked which static windows provide the best agreement between simplified ratios and kinetic modelling of the full dynamic PET scan. [^18^F]F-DED SUVr values were moderately to strongly positively associated with DVRs across all time windows for both AIE and MSA lesions (r^2^ = 0.48–0.91; *p* < 0.001; Fig. 4). The strongest DVR-to-SUVr agreement was observed between 30 and 50 min p.i. (for AIE: 30–40 min p.i.: r^2^ = 0.90, *p* < 0.0001; for MSA: 40–50 min p.i.: r^2^ = 0.91, *p* < 0.0001). The weakest agreement between DVR and SUVr was observed during the earliest 0–10 min window p.i. for both AIE (r^2^ = 0.51, *p* < 0.001) and MSA lesions (r^2^ = 0.48, *p* = 0.09). DVR and SUVr were congruently aligned in controls for temporal and frontal target regions, whereas DVR were slightly underestimated by SUVr for subcortical target regions in controls (Fig. 4).

### Discriminatory power of simplified and advanced quantification for the assessment of MAO-B expression in AIE and MSA lesions

**Fig. 5:**
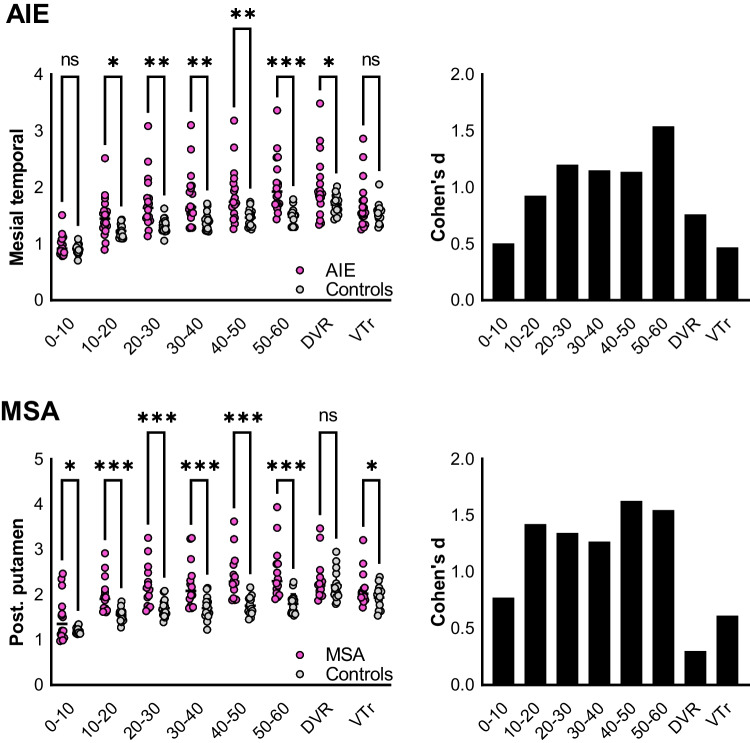
Discriminatory performance of simplified quantification in late imaging windows compared to kinetic modelling of dynamic [^18^F]F-DED PET – Scatter plots show [^18^F]F-DED PET signals derived from mesial temporal lesions of patients with autoimmune encephalitis (AIE, *n* = 18) and posterior putamen lesions of patients with multiple system atrophy (MSA, *n* = 14) compared to corresponding healthy tissue of controls. Multiple unpaired t-tests with false discovery rate correction were applied. Effect sizes for lesion in patients vs. healthy tissue in controls are shown for SUVr (0–10, 10–20, 20–30, 30–40, 40–50, 50–60 min p.i.), reference tissue modeling (DVR) and volume of distribution ratios (VTr). Of note, controls were on average older than patients, which may attenuate the observed differences, given that MAO-B expression can increase with age

Finally, we questioned which quantitative [^18^F]F-DED metrics provide optimal discrimination of MAO-B expression in lesions compared to healthy tissue. The discriminatory power was investigated by comparing differences and effect sizes for SUVr of different time frames, DVR, and VTr within the mesial temporal lobe (MTL, *n* = 18) for AIE and the posterior putamen (pPUT, *n* = 14) for MSA-P/C, versus controls respectively. Late-phase SUVr (30–40, 40–50, 50–60) were superior compared to DVR and VTr for the discrimination of AIE lesions from healthy tissue (Cohen’s d: 1.1–1.5 vs. 0.5–0.8) and for the discrimination of MSA lesions from healthy tissue (Cohen’s d: 1.3–1.6 vs. 0.3–0.6; Fig. 5). The superior discrimination was mainly driven by lower SUVr compared to DVR in healthy tissue during late-phase [^18^F]F-DED imaging.

## Discussion

In this well-characterized cohort of patients with autoimmune encephalitis (AIE), multiple system atrophy (MSA) and controls, we investigated the feasibility of short static acquisition protocols for [^18^F]F-DED PET imaging to detect reactive astrogliosis via targeting of MAO-B.

Our results demonstrate that even short 10-min late-phase recordings of [^18^F]F-DED PET are sufficient for detecting elevated MAO-B expression, correlating with previously investigated full 60-min dynamic PET scans [24]. In late imaging time frames the tracer uptake was consistently moderate to strong in disease-related target regions, predominantly including the mesial temporal lobe (MTL) for AIE and the posterior putamen (pPUT) and the cerebellar white matter (WM) for MSA-P/C [41–46]. SUVrs derived from early imaging (0–10 and 10–20 min p.i.) were significantly lower compared to SUVrs of all later time windows (20–30, 30–40, 40–50 and 50–60 min p.i.) in both AIE and MSA (average Cohen’s d for AIE: 1.69, average Cohen’s d for MSA: 1.16). SUVrs of late short static [^18^F]F-DED PET within 30 and 60 min p.i. showed the strongest signals and SUVrs as a function of time approached a plateau around 60 min p.i.. This pattern was aligned with the expected kinetic behavior (i.e. washout in regions with low MAO-B expression, but irreversible binding in regions with high MAO-B expression [24]) of [^18^F]F-DED and supported the suitability of later acquisition windows for detection of reactive astrogliosis.

Though SUVrs are more prone to the wash-in and washout of the tracer [47], [^18^F]F-DED SUVrs within 10 to 60 min p.i. were strongly associated with the 60-min full dynamic DVRs. In particular, the strongest agreement between SUVr and DVR was observed at 30–40-min p.i. in AIE (r^2^ = 0.90) and 40–50-min p.i. in MSA (r^2^ = 0.91), suggesting these intervals as optimal windows for standardized short static imaging and simplified quantification. A closely matching line of identity between SUVr and DVR supported no significant over or underestimation of tracer signals for 30–40-min and 40–50-min p.i. windows. Conversely, 0–10 SUVrs showed weak agreement with 0–60 DVRs, especially in MSA, where statistical significance was not reached (p = 0.09). Moreover, in AIE cases, SUVrs 0–10 even transiently ranged < 1.0, and similar values were also observed in controls when measured in the MTL, primarily suggesting a regional-dependent effect and potentially only secondarily reflecting hypoperfusion, maybe linked to disease-related changes. These findings point towards the potential for assessment of perfusion-related changes by early-phase [^18^F]F-DED imaging, but underline the preference for later static windows to detect reactive astrogliosis by [^18^F]F-DED PET. The ability to extract early-phase data from [^18^F]F-DED PET imaging as a surrogate of neuronal injury needs to be investigated in dedicated studies, as exemplarily presented for tau and β-amyloid radiotracers [48, 49]. While late short static SUVrs offer a pragmatic approximation to quantification, dynamic imaging still offers certain advantages, such as increased robustness against cerebral blood flow variations [50, 51]. Nevertheless, for routine diagnostic purposes of disabled patients, shorter static protocols can provide sufficient accuracy, balancing scan duration, data quality and patient comfort. In summary, our SUVr and DVR analysis provides promising evidence that 30–50 min is an ideal short imaging time window for [^18^F]F-DED PET since it reflects kinetic modeling data very well and catches most of the effect that is observed in later imaging windows. Although our data demonstrated a strong association between 0–60 min DVR and SUVr, we observed a better discrimination of lesion signals from healthy tissue using SUVr of late imaging time points compared to kinetic modelling parameters (DVR, VTr) of the full 60-min dynamic scan. These differences between SUVr, DVR, and VTr are likely depending on the kinetic profile of the tracer, reflecting varying regional physiological MAO-B expression and non-specific binding [18, 33, 34]. Specifically, the slight underestimation of DVR by SUVr is consistent with data of [^11^C]L-deprenyl-D2 PET, where simplified ratios (SUVr) did not fully capture the distribution volume at equilibrium as accurately as full kinetic modelling in regions with low MAO-B activity [52]. As a potential explanation, the faster wash-out relative to the specific binding rate in healthy tissue creates a 'low-signal' environment, where individual physiological variability in blood flow and non-specific binding show more relative contribution to the overall signal. Contrary, in the presence of high MAO-B expression (AIE and MSA lesions), the specific signal dominates the kinetic profile, leading to the robust SUVr-to-DVR agreement as observed in our dataset. In this regard, wash-out of the tracer from healthy tissue in controls during the late imaging phase facilitates detection of larger differences for lesion SUVr of patients with AIE and MSA.

All quantitative metrics were exploratively assessed using the cerebellar gray matter as a pseudo-reference region [19, 24]. Although this approach may have limitations in MSA-C due to potential confounding from variable MAO-B expression in cerebellar white matter [30, 31], our preliminary analysis did not show significant differences in cerebellar GM SUV between patients and controls. Interestingly, there was a trend towards lower [^18^F]F-DED SUV most pronounced in the early phase (0–10 min) for the MSA group compared to patients with AIE and controls. This may reflect lower tracer delivery, linked to cerebellar neurodegeneration and atrophy in MSA. However, since the cerebellar GM signal was not statistically different and comparable across groups in later time windows, the cerebellar GM was maintained as a reference region for the current study. Nonetheless, we highlight the need for future validation using a true arterial input function to definitively decouple specific binding from perfusion-related effects in these populations.

In addition, several other limitations of our study need to be considered. First, recommendations derived from our study are based on cross-sectional data and should be interpreted accordingly. Longitudinal validation and further clarification of [^18^F]F-DED PET as a neuroinflammation biomarker are still needed. Future studies should also investigate the phenotype-specific differences in radioligand clearance. Still, our data provide preliminary evidence that short static acquisition protocols with [^18^F]F-DED PET, particularly within the 30–50 min p.i, offer reliable detection of neuroinflammation and reactive astrogliosis while enhancing clinical feasibility and patient comfort, reinforcing the value of MAO-B PET as a biomarker for disease activity in conditions like AIE and MSA. Second, patients included in this study were imaged in clinical settings; therefore, an age-matched control cohort was not available. However, although age-related changes in MAO-B expression are known [34, 53], they do not likely confound the results of this study, since data were primarily compared between different quantification methods and not between patients and controls. As a limitation, we note that the differences in MAO-B between patients and controls could be attenuated since MAO-B expression and respective PET signals may increase as a function of age [13]. Future research should focus on validating these findings against quantification with arterial blood sampling and in additional patient cohorts with global (vs. lesion) changes in MAO-B expression.

## Conclusion

Our data support the feasibility of short static [^18^F]F-DED PET imaging for the detection of reactive astrogliosis in lesions of patients with AIE and MSA, which is particularly advantageous in neurologically severely affected patients. A late static acquisition between 30 and 50 min p.i. offers the best trade-off between accuracy and scanning efficiency when compared to kinetic modelling.

## Supplementary Information

Below is the link to the electronic supplementary material.

Supplementary file1 (DOCX 913 KB)

## Data Availability

The data supporting the findings of this study are available upon reasonable request from the corresponding author. The data are not publicly available due to privacy or ethical restrictions.
