# Supplementary material for: Feasibility of short imaging protocols for [18F]fluordeprenyl-D2 PET in autoimmune encephalitis and multiple system atrophy
Source: Eur J Nucl Med Mol Imaging. 2026 May 28;53(10):5709–20. doi: 10.1007/s00259-026-07932-0 (PMC13421227; doi:10.1007/s00259-026-07932-0)
Supplement: Supplementary file 1 — Supplementary file1 (DOCX 913 KB) [file 259_2026_7932_MOESM1_ESM.docx]

**Feasibility of Short Imaging Protocols for [^18^F]fluordeprenyl-D2 PET in Autoimmune Encephalitis and Multiple System Atrophy**

Lisa Tagnin^1^, Julia S. Dorneich^1^, Marianthi Zeinaki^1^, Letizia Vogler^1^, Laura Sanzo^1^, Johannes S. Gnörich^1,2^, Sabrina Katzdobler^2,3^, Ilias Masouris^2^, Alexander Jäck^2,3^, Alexander M. Bernhardt^3^, Boris-Stephan Rauchmann^4,5^, Sophia Stoecklein^6^, Marcel Simmet^1^, Emanuel Joseph^1^, Simon Lindner^1^, Norman Koglin^7^, Andre Mueller^7^, Andrew W. Stephens^7^, Gérard N. Bischof^7^, Lukas K. Frontzkowski^1,8^, Nicolai Franzmeier^1,8,9^, Rudolf A. Werner^1,10^, Jonathan A. Gernert^11^, Franziska Hopfner^2,3^, Günter U. Höglinger^2,3,12^, Robert Perneczky^2,5,12,13,14^, Carolin Kurz^5,15^, Tania Kümpfel^11,16^, Martin Kerschensteiner^11,12,16^, Franziska S. Thaler^11,16^, Johannes Levin^2,3,12^, Matthias Brendel^1,2,12*^

^1^Department of Nuclear Medicine, University Hospital of Munich, LMU Munich, Munich, Germany
^2^German Center for Neurodegenerative Diseases (DZNE), Munich, Germany

^3^Department of Neurology, University Hospital of Munich, LMU Munich, Munich, Germany

^4^Department of Neuroradiology, University Hospital of Munich, LMU Munich, Munich, Germany

^5^Department of Psychiatry and Psychotherapy, University Hospital of Munich, LMU Munich, Munich, Germany

^6^Department of Radiology, University Hospital of Munich, LMU Munich, Munich, Germany

^7^Life Molecular Imaging GmbH, a Lantheus Company, Berlin, Germany

^8^Institute for Stroke and Dementia Research, LMU Hospital, LMU Munich, Munich, Germany

^9^Department of Psychiatry and Neurochemistry, Institute of Neuroscience and Physiology, The Sahlgrenska Academy, University of Gothenburg, Mölndal, Gothenburg, Sweden

^10^Russell H. Morgan Department of Radiology and Radiological Sciences, Johns Hopkins School of Medicine, Baltimore, MD, U.S.A

^11^Institute of Clinical Neuroimmunology, University Hospital, LMU Munich, Munich, Germany

^12^Munich Cluster for Systems Neurology (SyNergy), Munich, Germany

^13^Ageing Epidemiology (AGE) Research Unit, School of Public Health, Imperial College, London, UK

^14^Sheffield Institute for Translational Neuroscience (SITraN), University of Sheffield, Sheffield, UK

^15^Department of Psychiatry and Psychotherapy, Technical University of Munich, TUM School of Medicine and Health, TUM University Hospital, Munich, Germany

^16^Biomedical Center, Faculty of Medicine, LMU Munich, Munich, Germany

**Corresponding author:**

Dr. Matthias Brendel

Department of Nuclear Medicine, University of Munich

Marchioninstraße 15, 81377 Munich, Germany

E-Mail: Matthias.Brendel@med.uni-muenchen.de

**Supplemental Figure 1:** Comparison of SUV of the cerebellar grey matter across time windows (Welch’s test corrected by FDR). CTRL = controls, AIE = autoimmune encephalitis, MSA = multiple system atrophy


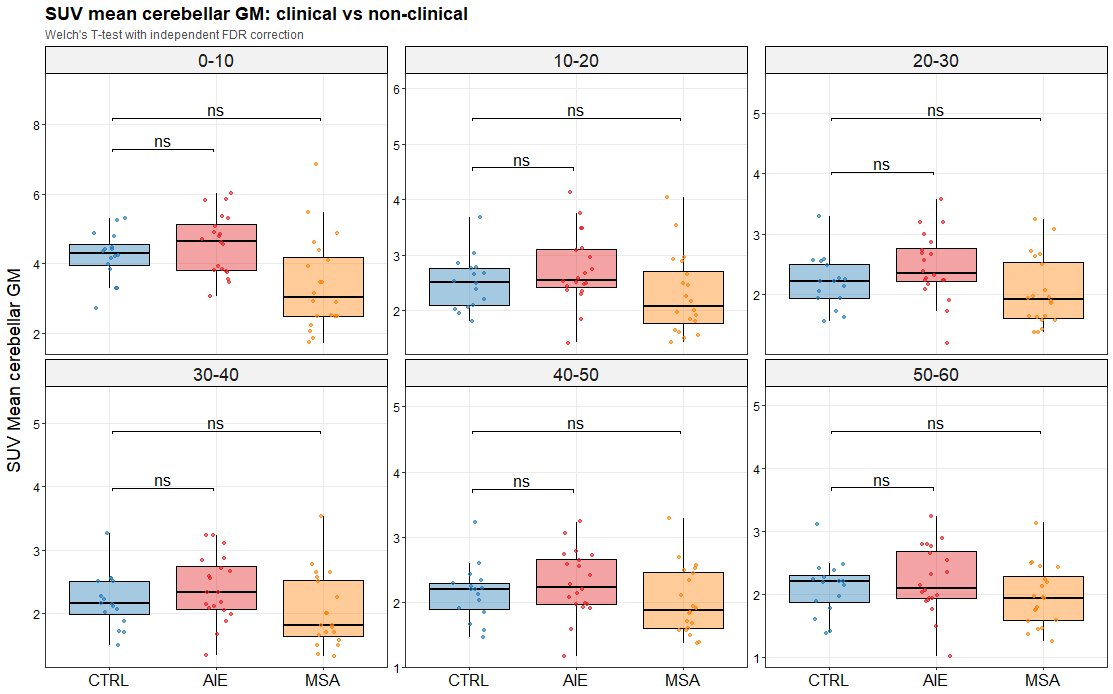


**Supplemental Figure 2:** Visual comparison of signal to noise across 30-60, 40-60, 50-60 and 55-60 minutes p.i.


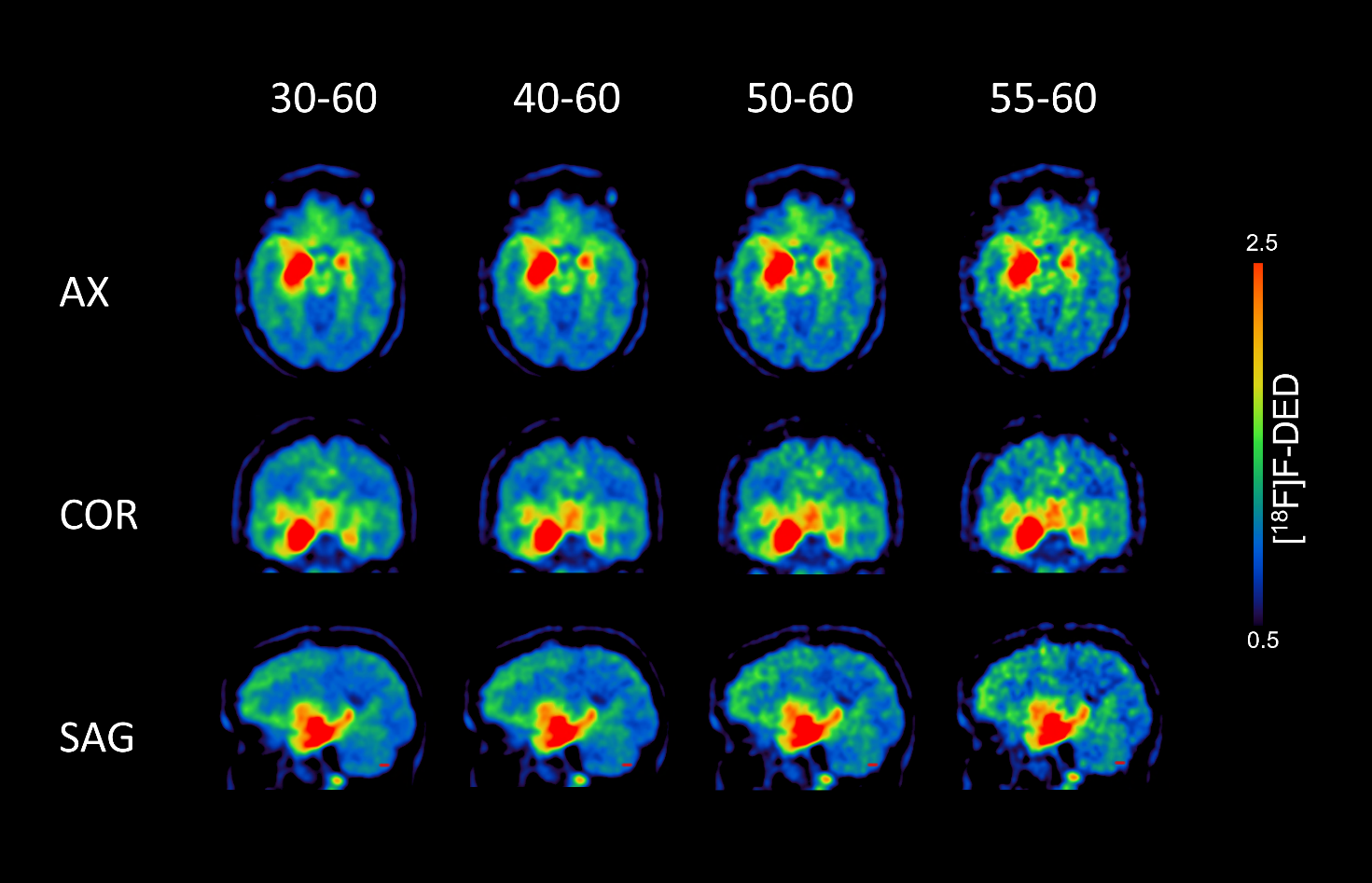


**Supplemental Table 1:** Comparison of SUVr across time windows (mixed ANOVA corrected by Tukey’s post hoc test) for AIE lesions

| Mixed ANOVA Results for SUVr Mean in AIE | | | | | | | | |
| --- | --- | --- | --- | --- | --- | --- | --- | --- |
| Analysis of SUVr Mean Across 10-min intervals from 0 to 60 min p.i. | | | | | | | | |
| Effect | DFn | DFd | SSB | SSW | F-value | p-value | < 0.05 | ges |
| Intercept | 1 | 19 | 320.37 | 16.46 | 369.88 | 6.48e-14 | * | 0.95 |
| Time windows | 5 | 95 | 13.37 | 2.65 | 95.93 | 1.46e-35 | * | 0.83 |

| Tukey's Test post-hoc for SUVr mean in AIE | | | | | | |
| --- | --- | --- | --- | --- | --- | --- |
| SUVr mean across 10 min intervals time windows from 0 to 60 min p.i. | | | | | | |
| TW comparison | Mean diff | SE | DF | T-ratio | p-value | < 0.05 |
| 0-10 vs 10-20 | -0.49 | 0.05 | 95 | -9.19 | 4.64e-10 | * |
| 0-10 vs 20-30 | -0.73 | 0.05 | 95 | -13.87 | 4.63e-10 | * |
| 0-10 vs 30-40 | -0.81 | 0.05 | 95 | -15.31 | 4.63e-10 | * |
| 0-10 vs 40-50 | -0.86 | 0.05 | 95 | -16.30 | 4.63e-10 | * |
| 0-10 vs 50-60 | -1.03 | 0.05 | 95 | -19.47 | 4.63e-10 | * |
| 10-20 vs 20-30 | -0.25 | 0.05 | 95 | -4.68 | 1.36e-04 | * |
| 10-20 vs 30-40 | -0.32 | 0.05 | 95 | -6.11 | 3.20e-07 | * |
| 10-20 vs 40-50 | -0.38 | 0.05 | 95 | -7.10 | 3.77e-09 | * |
| 10-20 vs 50-60 | -0.54 | 0.05 | 95 | -10.28 | 4.64e-10 | * |
| 20-30 vs 30-40 | -0.08 | 0.05 | 95 | -1.43 | 7.07e-01 |  |
| 20-30 vs 40-50 | -0.13 | 0.05 | 95 | -2.42 | 1.59e-01 |  |
| 20-30 vs 50-60 | -0.30 | 0.05 | 95 | -5.59 | 3.13e-06 | * |
| 30-40 vs 40-50 | -0.05 | 0.05 | 95 | -0.99 | 9.20e-01 |  |
| 30-40 vs 50-60 | -0.22 | 0.05 | 95 | -4.16 | 9.54e-04 | * |
| 40-50 vs 50-60 | -0.17 | 0.05 | 95 | -3.17 | 2.42e-02 | * |

**Supplemental Table 2:** Effect sizes for comparison of SUVr across time windows in AIE

| TW comparison | Cohen's d | Lower CI | Upper CI | Effect size |
| --- | --- | --- | --- | --- |
| 10-20 vs 0-10 | 1.779 | 1.023 | 2.535 | large |
| 20-30 vs 0-10 | 2.153 | 1.349 | 2.958 | large |
| 30-40 vs 0-10 | 2.313 | 1.486 | 3.140 | large |
| 40-50 vs 0-10 | 2.456 | 1.608 | 3.304 | large |
| 50-60 vs 0-10 | 2.895 | 1.979 | 3.811 | large |
| 20-30 vs 10-20 | 0.616 | 0.039 | 1.271 | moderate |
| 30-40 vs 10-20 | 0.789 | 0.124 | 1.454 | moderate |
| 40-50 vs 10-20 | 0.915 | 0.242 | 1.588 | large |
| 50-60 vs 10-20 | 1.311 | 0.605 | 2.016 | large |
| 30-40 vs 20-30 | 0.166 | 0.476 | 0.807 | no effect |
| 40-50 vs 20-30 | 0.280 | 0.364 | 0.923 | small |
| 50-60 vs 20-30 | 0.641 | 0.016 | 1.297 | moderate |
| 40-50 vs 30-40 | 0.113 | 0.528 | 0.753 | no effect |
| 50-6 vs 30-40 | 0.470 | 0.179 | 1.119 | small |
| 50-60 vs 40-5 | 0.357 | 0.288 | 1.003 | small |

**Supplemental Table 3:** Comparison of SUVr across time windows (mixed ANOVA corrected by Tukey’s post hoc test) for MSA lesions

| Mixed ANOVA Results for SUVr Mean in MSA | | | | | | | | |
| --- | --- | --- | --- | --- | --- | --- | --- | --- |
| Analysis of SUVr Mean Across 10-min intervals from 0 to 60 min p.i. | | | | | | | | |
| Effect | DFn | DFd | SSB | SSW | F-value | p-value | < 0.05 | ges |
| Intercept | 1 | 19 | 505.57 | 23.42 | 410.20 | 2.53e-14 | * | 0.96 |
| Time windows | 5 | 95 | 11.30 | 3.42 | 62.75 | 1.38e-28 | * | 0.77 |

| Tukey's Test post-hoc for SUVr mean in MSA | | | | | | |
| --- | --- | --- | --- | --- | --- | --- |
| SUVr mean across 10 min intervals time windows from 0 to 60 min p.i. | | | | | | |
| TW comparison | Mean diff | SE | DF | T-ratio | p-value | < 0.05 |
| 0-10 vs 10-20 | -0.47 | 0.06 | 95 | -7.86 | 5.52e-10 | * |
| 0-10 vs 20-30 | -0.68 | 0.06 | 95 | -11.27 | 4.63e-10 | * |
| 0-10 vs 30-40 | -0.72 | 0.06 | 95 | -11.94 | 4.63e-10 | * |
| 0-10 vs 40-50 | -0.82 | 0.06 | 95 | -13.66 | 4.63e-10 | * |
| 0-10 vs 50-60 | -0.95 | 0.06 | 95 | -15.80 | 4.63e-10 | * |
| 10-20 vs 20-30 | -0.20 | 0.06 | 95 | -3.41 | 1.20e-02 | * |
| 10-20 vs 30-40 | -0.24 | 0.06 | 95 | -4.07 | 1.31e-03 | * |
| 10-20 vs 40-50 | -0.35 | 0.06 | 95 | -5.79 | 1.31e-06 | * |
| 10-20 vs 50-60 | -0.48 | 0.06 | 95 | -7.94 | 5.25e-10 | * |
| 20-30 vs 30-40 | -0.04 | 0.06 | 95 | -0.67 | 9.85e-01 |  |
| 20-30 vs 40-50 | -0.14 | 0.06 | 95 | -2.39 | 1.72e-01 |  |
| 20-30 vs 50-60 | -0.27 | 0.06 | 95 | -4.53 | 2.45e-04 | * |
| 30-40 vs 40-50 | -0.10 | 0.06 | 95 | -1.72 | 5.22e-01 |  |
| 30-40 vs 50-60 | -0.23 | 0.06 | 95 | -3.86 | 2.75e-03 | * |
| 40-50 vs 50-60 | -0.13 | 0.06 | 95 | -2.14 | 2.75e-01 |  |

**Supplemental Table 4:** Effect sizes for comparison of SUVr across time windows in MSA

| TW comparison | Cohen's d | Lower CI | Upper CI | Effect size |
| --- | --- | --- | --- | --- |
| 10-20 vs 0-10 | 1.148 | 0.457 | 1.839 | large |
| 20-30 vs 0-10 | 1.462 | 0.741 | 2.183 | large |
| 30-40 vs 0-10 | 1.503 | 0.778 | 2.227 | large |
| 40-50 vs 0-10 | 1.683 | 0.938 | 2.428 | large |
| 50-60 vs 0-10 | 1.819 | 1.058 | 2.580 | large |
| 20-30 vs 10-20 | 0.487 | 0.163 | 1.137 | small |
| 30-40 vs 10-20 | 0.561 | 0.091 | 1.214 | moderate |
| 40-50 vs 10-20 | 0.778 | 0.114 | 1.442 | moderate |
| 50-60 vs 10-20 | 0.984 | 0.307 | 1.662 | large |
| 30-40 vs 20-30 | 0.082 | 0.558 | 0.723 | no effect |
| 40-50 vs 20-30 | 0.289 | 0.354 | 0.933 | small |
| 50-60 vs 20-30 | 0.514 | 0.136 | 1.165 | moderate |
| 40-50 vs 30-40 | 0.203 | 0.439 | 0.845 | small |
| 50-60 vs 30-40 | 0.429 | 0.219 | 1.076 | small |
| 50-60 vs 40-50 | 0.234 | 0.409 | 0.876 | small |
